# Measuring Cognitive Impairments Associated With Schizophrenia in Clinical Practice: Overview of Current Challenges and Future Opportunities

**DOI:** 10.1093/schbul/sbae051

**Published:** 2024-08-01

**Authors:** Keith H Nuechterlein, Henry Nasrallah, Dawn Velligan

**Affiliations:** Department of Psychiatry and Biobehavioral Sciences, University of California Los Angeles, Los Angeles, CA; Department of Psychiatry and Behavioral Neuroscience, University of Cincinnati School of Medicine, Cincinnati, OH; Division of Schizophrenia and Related Disorders, Department of Psychiatry and Behavioral Sciences, University of Texas Health Science Center, San Antonio, TX

**Keywords:** cost-effective cognitive assessment, brief cognitive assessment, clinician training, performance-based assessment, interview-based assessment

## Abstract

**Background:**

Cognitive impairment associated with schizophrenia (CIAS) negatively impacts daily functioning, quality of life, and recovery, yet effective pharmacotherapies and practical assessments for clinical practice are lacking. Despite the pivotal progress made with establishment of the Measurement and Treatment Research to Improve Cognition in Schizophrenia (MATRICS) Consensus Cognitive Battery (MCCB) for clinical research, implementation of the full MCCB is too time-consuming and cost-ineffective for most clinicians in clinical practice.

**Study Design:**

Here we discuss current assessments in relation to delivery format (interview-based and performance-based), validity, ease of use for clinicians and patients, reliability/reproducibility, cost-effectiveness, and suitability for clinical implementation. Key challenges and future opportunities for improving cognitive assessments are also presented.

**Study results:**

Current assessments that require 30 min to complete would have value in clinical settings, but the associated staff training and time required might preclude their application in most clinical settings. Initial profiling of cognitive deficits may require about 30 min to assist in the selection of evidence-based treatments; follow-up monitoring with brief assessments (10–15 min in duration) to detect treatment-related effects on global cognition may complement this approach. Guidance on validated brief cognitive tests for the strategic monitoring of treatment effects on CIAS is necessary.

**Conclusions:**

With increased advancements in technology-based and remote assessments, development of validated formats of remote and in-person assessment, and the necessary training models and infrastructure required for implementation, are likely to be of increasing clinical relevance for future clinical practice.

## Introduction

Cognitive deficits constitute one of the main limiting factors for recovery in the context of treatment and rehabilitation in schizophrenia, yet no effective pharmacotherapies targeting cognition are currently available.^1,2^ Impaired cognition is reported not only in schizophrenia, but also across a number of other psychiatric conditions including major depressive disorder, bipolar disorder, and posttraumatic stress disorder.^3,4^ The symptom overlap, and similarity in the patterns of redundant neurocircuitry associated with cognitive impairments across these conditions, suggest possible shared pathological mechanisms underpinning these deficits.^3,5^

In schizophrenia, cognitive impairments have come to be recognized as the most prominent factors limiting daily work/school and social functioning and the quality of life of patients.^6–12^ They also contribute significantly to the financial burden related to this disorder.^13^ Recognition of the importance of cognitive impairments in limiting functional recovery led the National Institute of Mental Health (NIMH) to bring focused attention to cognition as an area in which new treatment development was a high priority.^14,15^ This increased focus on the development and evaluation of improved treatments for cognitive impairments in schizophrenia provides further impetus to improve cognitive assessments and increase awareness among the clinical community of the importance of addressing these features.^16–18^ If we hope to address cognitive impairments in schizophrenia and thereby improve functional outcomes, increased emphasis on detecting and assessing cognitive deficits in clinical practice is necessary.

Detection of cognitive impairments by clinicians is often hindered by limited patient self-reporting of these deficits that can result from the lack of insight into illness, reduced motivation, and stigma experienced by people with schizophrenia and psychotic disorders.^19–23^ These additional challenges faced by patients, in turn, contribute to the high risk of nonadherence to treatments, negative clinical outcomes, and reduced functioning.^19–23^ To achieve positive treatment attitudes and therapeutic alliance with patients, and thus improve patient self-reporting of cognitive deficits, it is important to enhance psychoeducation, effectively communicate with patients and their care-givers, and include patients in the treatment decision process.^24,25^

In addition to identifying cognitive deficits, healthcare providers face a number of additional challenges in treating these deficits, including: a poor understanding of cognitive impairments and their assessment, a lack of patient insight into their cognitive deficits, a lack of clear guidance on available cognitive assessments and treatments, and logistical barriers such as constraints on the time, training and resources needed for clinicians to administer available assessments within healthcare systems.^19,23,26,27^ OnTrackNY recently developed a toolkit to help clinicians assess and address cognitive health in patients with early psychosis.^28^ In a study evaluating this toolkit, over 50% of the 933 participants assessed (young people who had experienced a first episode of non-affective psychosis) self-reported cognitive problems. The decision-making tools and assessments were shown to successfully assist with the management of cognitive deficits.^28^ An opportunity for clinicians to customize cognitive assessments and treatments to individual patients based on the nature of presenting problems is also supported by recent evidence that separable aspects of cognition, such as neurocognition and social cognition, predict different functional outcomes.^7^

Cognitive deficits are a serious component of schizophrenia that should be evaluated and treated by clinicians using evidence-based pharmacological, somatic, and psychological therapies. Current treatments that represent promising treatment strategies for cognitive dysfunction include psychosocial and neuromodulatory interventions such as cognitive remediation training (CRT), cognitive adaptation training, transcranial magnetic stimulation (TMS), and transcranial direct current stimulation (tDCS).^29–32^ Recent metanalyses have shown that the benefits of these treatments appear to selectively impact on specific cognitive domains.^32,33^ As clinicians attempt to incorporate new and existing treatments for cognitive deficits into their repertoire of interventions, improved cognitive assessment will be needed to identify the severity of cognitive deficits in individual patients, the pattern of deficits across cognitive domains, and their changes over the course of treatment. In theory, clinical assessments that provide accurate cognitive profiles of patients in the clinic may allow mental healthcare providers to streamline treatment approaches, and align patients presenting with impairments in specific cognitive domains with treatments that target these impairments.

Interestingly, the most commonly employed cognitive assessment tool used to measure current treatment effects on cognition in two recent meta-analysis studies was the Measurement and Treatment Research to Improve Cognition in Schizophrenia (MATRICS) Consensus Cognitive Battery (MCCB) followed by the Brief Assessment of Cognition in Schizophrenia (BACS).^32,33^ Although the full MCCB is an excellent, validated assessment for measuring cognition in research contexts, the cost and staff time and training required for its implementation make it logistically difficult to use for the purpose of continued monitoring of cognitive changes in real-world clinical settings. The BACS is much shorter, about 30 min, but clinicians may still find it too long for repeated clinical monitoring of cognition. These challenges might also contribute to the reduced referral rates for CRT from clinical versus research sites,^34^ highlighting the need for improved assessment strategies in clinical settings. The ultimate goal is to equip clinicians with the best assessment methods, and guidance for their implementation, so that they in turn may better guide, motivate, and educate patients on the most appropriate therapies to help manage their cognitive impairments. A critical first step is the refinement of the assessment process, so that cognitive deficits can be accurately identified, characterized, and monitored during the course of treatment.

The MATRICS initiative was launched in 2002 by the NIMH^35^ to address the urgent need to improve understanding of cognitive neurobiology and to develop enhanced and effective assessment methods to evaluate cognitive treatments in schizophrenia. The MATRICS initiative involved strategic discussions with experts from relevant fields in academia, the Food and Drug Administration, NIMH, and the pharmaceutical industry to address the lack of consensus that existed regarding how cognition is best assessed, both in relation to specific clinical tests administered, and the broad spectrum of cognitive domains affected.^35,36^ These collaborative meetings and associated empirical studies identified key cognitive domains to be captured in assessment batteries for cognitive impairment associated with schizophrenia (CIAS) and related disorders, and outlined the optimal methods for cognitive assessment that paved the way forward for effective treatments.^9,35,36^ The product of expert discussions and empirical comparisons of promising measures was the development of the MCCB that consists of recommended cognitive assessments and includes a standardized computerized scoring system to be adopted by studies evaluating novel compounds for CIAS.^36,37^

Despite this pivotal initial step along the progressive path to improved assessments for CIAS, and the remarkable benefits provided by the MCCB for clinical research, use of the MCCB in everyday clinical practice can be limited due to logistical reasons such as lack of trained staff, and the amount of time required to administer (approximately 65–90 min). Research initiatives and funding calls to develop improved cognitive assessments and treatments have increased in recent years.^16–18^ Although a transdiagnostic approach to the investigation of cognitive impairments across psychiatric disorders is supported by the recent literature,^38^ an examination of cognitive assessments for clinical practice for other major psychiatric disorders is beyond the scope of this review. Here, we focus on CIAS, and the suitability of current clinical assessments for CIAS in everyday clinical practice. We aim to highlight the unmet need in assessment of cognition and functioning in clinical practice and discuss current assessments in relation to validity, ease of use for clinicians and patients, reliability/reproducibility, and cost-effectiveness. Finally, the main challenges and future opportunities for improving and facilitating assessments will be discussed.

## Cognitive Domains in Schizophrenia Spectrum and Other Psychotic Disorders

Recent years have seen major progress in our understanding of the underlying cognitive pathology of schizophrenia, including delineating specific domains outlined in the MATRICS initiative for neurocognition and social cognition.^39,40^ CIAS represents a core feature of these disorders and is reported in approximately 60%–98% of patients with schizophrenia and psychotic disorders.^41–43^ Meta-analyses have demonstrated that both the qualitative and quantitative nature and the temporal pattern of CIAS vary, including impacts on neurocognitive domains that differ in the severity of impairments and their occurrence along the course of the prodromal phase of illness.^44–47^

The MCCB includes assessment of seven cognitive domains in total; these are speed of processing, attention/vigilance, working memory, verbal learning, visual learning, reasoning and problem solving, and social cognition.^36^ Although the number of separable cognitive dimensions in schizophrenia has been debated, findings support use of this seven-factor model for clinical trials seeking interventions to improve cognition in schizophrenia.^9,48^

Social cognition, defined as cognitive processes needed to perceive, interpret, and process information for adaptive social interactions,^6,49^ has been shown to be interlinked with neurocognition and daily functioning.^50,51^ Deficits in social cognition are well characterized in schizophrenia and limit functional recovery.^49,50,52^ However, a better understanding of social cognition and its neurobiological correlates in schizophrenia is needed to improve assessments to evaluate new effective therapies for social disability in this complex disorder.^49,53^

## Interview-based Cognitive Assessments

In clinical settings, the required expertise or resources to conduct and interpret performance-based measures is not always accessible to clinicians and may lead to a preference for different approaches or supplemental assessments; interview-based assessments provide promising alternatives.^54^

There are specific advantages of interview-based assessments of cognitive functioning, including their ease of use and capacity to consider patient/informant reports of impact on daily functioning.^55^ Factoring in the patient perspective and comparing patient self-assessment with informant-assessment of cognitive abilities provides an important means of examining neurocognitive insight in patients with schizophrenia.^54^

A limitation of interview-based cognitive assessments is that they require insight into cognitive ability for accuracy, and this insight is often absent or incomplete in patients with schizophrenia spectrum disorders.^19,56^ Poor insight into illness (anosognosia) is exhibited by 57%–98% of patients with schizophrenia spectrum disorders and is characterized by a lack of awareness of having a psychiatric illness, or cognitive appraisal of one’s own state or the need for treatment.^19,57^ Anosognosia leads to poorer quality of life and functioning and also impacts medication adherence and the reliability of self-report assessments,^19,58^ highlighting the importance of identifying and measuring anosognosia to optimize clinical decisions relating to assessment and therapy. A related limitation is that reporting one’s cognitive deficits requires a certain level of cognitive functioning (eg, memory of one’s memory problems). Finally, another limitation of interview-based assessments is the variation in reported correlations between interview-based and performance-based cognitive assessments. Some studies demonstrate positive correlations (though of limited magnitude),^55,56,59–61^ while others report no or minimal correlation,^59,62^ suggesting self-reports alone might have limited validity, but can add value when administered with performance-based assessments. Use of informant reports increases validity but makes these assessments less convenient.^63^

Examples of validated interview assessments that include both patient and informant reports in their overall measures are the Schizophrenia Cognitive Rating Scale (SCoRS) and the Cognitive Assessment Interview (CAI).^54,61^ With many similar advantages including their moderately short administration times (SCoRS, 25–35 min; CAI, 30–35 min, Table 1), breadth of assessment across cognitive domains, validation in diverse languages and cultures, their simplicity, test–retest reliability, high degree of correlation with functional outcome measures, and established correlation with cognitive performance measures, the SCoRS and CAI provide promising options for cognitive assessment in the clinic.^61,64–67^ The SCoRS is also recommended as a co-primary measure of cognition alongside cognitive test batteries in clinical trials.^64,68^ Despite these advantages, advanced rater training is still required for administration and scoring of both the SCoRS and CAI,^61,68^ and for the SCoRS, considerable geographical variability exists, and varied psychometric properties across clinical trial sites in accordance with rater experience have been reported.^68^ Additionally, informant information is not always available for patients, and can vary in quality depending on how well informants understand that cognition impacts daily functioning, presenting additional challenges in the implementation and interpretation of these test results by clinicians.^56,68,69^ The need for simplified formats that minimize training times is essential. As the CAI was originally developed to form a combined abbreviated version of the Clinical Global Impression of Cognition in Schizophrenia and the SCoRS assessments, it is relatively easy to administer, score, and interpret, with minimal practice effects, making it suitable option for repeated administration in the monitoring of treatment effects.^61^A summary of the main interview-based cognitive assessments currently in use is provided in Table 1.

**Table 1.** Summary of Interview-based Assessments Currently Used to Measure CIAS and Their Applicability to Clinical Settings

Interview-based Assessments							
Test	Cognitive Domain(s) Assessed	Availability to Clinicians (Computerized Version Available; √/x)	Description	Estimated Completion: Scoring Time^a^ (min)	Reporter	Suitability for Clinical Settings	
						Advantages	Disadvantages
CAI^61,70^	6 domains: • Processing speed • Attention/vigilance • Working memory • Verbal learning • Reasoning/problem solving • Social cognition	Paper version free (✓)	10-item interview; rater scores along a 7-point scale (patient and informant scores are combined)	30:5	• Patient • Informant	• Minimal practice effects • High item-to-scale correlations • Good test–retest reliability (ICC 0.79–0.84) • Established correlations with neurocognition and functional outcomes • Easily translatable and culturally adaptable • Incorporates patient perspective	• Rater training required • Reliant on informants that are not always available
MIC^71^	3 domains: • Attention • Working memory • Reasoning/problem solving	NA (✓)	MIC-clinician rated (MIC-CR): Clinicians complete a semi-structured interview on 12 cognitive tasks and rated on a 5-point scale MIC-self report (MIC-SR): Patient responds to 12 statements on cognitive ability and total scores range from 0 to 36	Unknown	• Patient • Professional staff member	• High correlations between MIC-CR and MIC-SR • Good retest reliability (ICC: 0.83–0.93) • Established correlations with psychiatric symptoms • Incorporates patient perspective	• Not well correlated with performance-based measures of cognition • Designed as measure of awareness of cognitive problems rather than a measure of cognitive deficits
SCoRS^54,64,68^	7 domains and motor skills: • Processing speed • Attention/vigilance • Working memory • Verbal learning • Visual learning • Reasoning/problem solving • Social cognition	Paper version free; computerized version requires license (✓)	20-item interview; rater scores along a 4-point scale based on judgment of all interviews to generate a global rating from 1 to 10	25:10	• Patient • Informant • Professional staff member	• Good test–retest reliability (ICC: > 0.80) • Established correlations with neurocognition and with functional outcomes • Sensitive to treatment effects • Relatively quick and easy administration, scoring and interpretation • Easily translatable and culturally adaptable • Incorporates patient perspective	• Rater training required • Reliant on informants that are not always available
B-CATS^6,72,73^	4 domains: • Attention/vigilance • Verbal learning • Visual learning • Reasoning/problem solving	Licensing fee applies (✓)	21-item interview; rater scores along a 50-point scale based on judgment of all interviews to generate a global rating from 1 to 10	10–20:2	• Patient • Professional staff member	• Assesses 4 domains to yield measure of global cognitive function • Excellent test–retest reliability (ICC:0.99) • Excellent internal consistency • Excellent construct and predictive validity • Minimal rater training required • Short Form version of the B-CATS is available	• Sensitive to severe–mild cognitive impairments in patients in assisted-living facilities but not validated in patients with schizophrenia • Does not allow any pattern of alterations to be evaluated

*Note:* B-CATS, Brief Cognitive Assessment Tool for Schizophrenia; CAI, Cognitive Assessment Interview; ICC, Intraclass Correlation; MIC, Measurement of Insight into Cognition scale; SCoRS, Schizophrenia Cognition Rating Scale.

^a^Scoring times are estimated based on author experience.

## Performance-based Cognitive Assessments

Performance-based assessments differ greatly in the length of administration and scoring time and the mode in which they are delivered; many are conducted on pen-and-paper while others involve computerized administration. A summary of current performance-based assessments for CIAS is presented in Tables 2 and 3. Here, we discuss tests of short and intermediate length, given the lack of feasibility of repeated assessments with the longer batteries in everyday clinical practice.

**Table 2.** Summary of Performance-based Assessments Currently Used to Measure CIAS Within the MCCB and Their Applicability to Clinical Settings

Performance-based Assessments as Part of the MCCB							
Test	Cognitive Domains Assessed	Availability to Clinicians (Computerized Version Available; ✓/✘)	Description	Estimated Completion: Scoring time^a^ (min)	Reporter	Suitability for Clinical Settings	
						Advantages	Disadvantages
• MATRICS consensus cognitive battery (MCCB)							
MCCB full assessment^8,9,36,37,74^	7 domains; • Processing speed • Attention/vigilance • Working memory • Verbal learning • Visual learning • Reasoning/problem solving • Social cognition	Distribution through Psychological Assessment Resources, Inc. (✘; some subtests and/or scoring are computerized)	10 tests administered by clinician **Outcome measure:** computer program generates individual test T-scores, cognitive domain T-score, neurocognitive composite T-score (non-social cognition) and overall composite T-score	60:10	Professional staff member	• Assesses global cognitive function and profile of individual cognitive domains • Good test–retest reliability (ICC: 0.88) • Good correlation with functional outcome and high tolerability by respondents • Standardized battery that allows comparison across institutions • Multiple demographic corrections are possible • Available in > 35 languages • Includes measure of social cognition	• Requires rater training (approx. 1 day) • Requires relatively long period of time to administer
BACS: symbol-coding^36^	Processing speed	Licensing fee (✓)	Participants assign numbers to non-meaningful symbols. **Outcome measure**: Items completed correctly within the 90 second test.	2:1	Professional staff member	• Brief testing time • Amenable to computerized delivery and scoring • Good test–retest reliability (ICC: 0.85)	• Limited to 1 cognitive domain when performed alone
Category fluency: animal naming^36^		Free (✓)	Respondents say as many animals as possible within 60 seconds. **Outcome measures:** Total animals named	1:1	Professional staff member	• Brief testing time • Amenable to computerized delivery and scoring • Good test–retest reliability (ICC: 0.74)	• Limited to 1 cognitive domain when performed alone
TMT: Part A^36^		Free (✘)	Timed paper-and-pencil test; Part A requires linking numbers in sequence as quickly as possible **Outcome measure:** number of correct attempts in allotted time	1:1	Professional staff member	• Brief testing time • Good test–retest reliability (ICC: 0.75)	• Limited to 1 cognitive domain when performed alone
CPT-IP^36^	Attention/vigilance	Licensing fee (✓)	Computerized test to identify identical stimulus pairs within a continuously presented series of number stimuli **Outcome measure:** target detection accuracy	12:1	Professional staff member	• Brief testing time • Computerized delivery and scoring • Good test–retest reliability (ICC: 0.84)	• Limited to 1 cognitive domain when performed alone
WMS^®^-III: Spatial Span^36^	Working memory (nonverbal)	Licensing fee (✓;computerized Cambridge Cognition Spatial Span Test)	Using a board on which 10 cubes are irregularly spaced, respondent taps cubes in same (or reverse) sequence as test administrator. **Outcome measure:** span length (the longest sequence successfully recalled), errors, number of attempts and latency (speed of response).	4:1	Professional staff member	• Brief testing time • Amenable to computerized delivery and scoring • Good test–retest reliability (ICC: 0.74)	• Limited to 1 cognitive domain when performed alone
Letter–Number Span^36^	Working memory (verbal)	Licensing fee (✓; computerized Cambridge Cognition Digit Span test)	Respondents hear a sequence of digits and letters and then recite by number order followed by letter order. **Outcome measure:** the longest sequence successfully reached, and the total attempts.	5:1	Professional staff member	• Brief testing time • Amenable to computerized delivery and scoring • Good test-re-test reliability (ICC: 0.78) • Strong relationship to global functional status	• Limited to 1 cognitive domain when performed alone
HVLT-R^36^	Verbal learning	Licensing fee (✘)	12 words from 3 taxonomic categories are orally presented and respondent recalls as many as possible after each of 3 learning trials. **Outcome measure:** raw scores derived for Total Recall	3:1	Professional staff member	• Brief testing time • Good test–retest reliability (ICC: 0.68) • Availability of six parallel forms may be helpful for clinical trials with multiple test occasions	• Limited to 1 cognitive domain when performed alone
BVMT-R^36^	Visual learning	Licensing fee (✘)	Participant reproduces six geometric figures from memory after 3 learning trials: stimulus viewed for 10 seconds. **Outcome measure**: raw scores for total recall	4:3	Professional staff member	• Brief testing time • Good test–retest reliability (ICC: 0.71) • Availability of six parallel forms	• Limited to 1 cognitive domain when performed alone
NAB: Mazes^36,75^	Reasoning and problem solving	Licensing fee (✘)	7 timed paper-and-pencil mazes of increasing difficulty to measure foresight and planning **Outcome measure:** total raw score	10:1	Professional staff member	• Brief testing time • Amenable to computerized delivery and scoring • Good test–retest reliability (ICC: 0.83)	• Limited to 1 cognitive domain when performed alone
MSCEIT: managing emotions^36^	Social cognition	Licensing fee (✘)	Paper-and-pencil multiple-choice test that assesses how people manage emotions of self and others **Outcome measure:** branch score using general consensus scoring	11:5	Professional staff member	• Brief testing time • Good test–retest reliability (ICC: 0.73) • Strong relationship to global functional status	• Limited to 1 cognitive domain when performed alone

*Note:* BACS, Brief Assessment of Cognition in Schizophrenia; BVMT-R, Brief Visuospatial Memory Test-Revised; CPT-IP, Continuous Performance Test—Identical Pairs; HVLT-R, Hopkins Verbal Learning Test-Revised; ICC, intraclass correlation; MSCEIT, Mayer–Salovey-5 Caruso Emotional Intelligence Test; NAB, Neuropsychological Assessment Battery; TMT, Trail Making Test; VFT, WMS-III, Wechsler Memory Scale-3rd Edition.

^a^Scoring times are estimated based on author experience.

**Table 3.** Summary of Performance-based Assessments Outside of MCCB Currently Used to Measure CIAS and Their Applicability to Clinical Settings

Other Performance-based Assessments that are not Components of the MCCB							
Test	Cognitive Domains Assessed	Availability to Clinicians (Computerized Version Available; ✓/✘	Description	Estimated Completion: Scoring^a^ Time (min)	Reporter	Suitability for Clinical Settings	
						Advantages	Disadvantages
CANTAB^76–78^	6 domains; • Processing speed • Attention/vigilance • Working memory • Visual learning • Reasoning/problem solving • Psychosocial functioning	Licensing fee; available from Cambridge Cognition (✓)	8 CANTAB tests provided for schizophrenia^b^ **Outcome measure:** individual task Z-scores and composite score	45:5	Professional staff member	• Computerized battery with standardized delivery • Sensitive to pharmacological and environmental effects in healthy and patient populations • Established correlations with neurocognition and with functional outcomes • Assesses multiple cognitive domains • No technical knowledge or training required • Language-independent • Has translational utility	• Relatively long administration time • Specialized computer equipment
BACS^79–81^	5 domains & Motor function; • Processing speed • Attention/vigilance • Working memory • Verbal learning • Reasoning/problem solving	Licensing fee; Available from WCG Clinical (✓)	6 BACS tests^c^ **Outcome measure:** individual task Z-scores and composite score	30–35:5 (longer scoring time for pen/paper version)	Professional staff member	• Assesses multiple domains to yield a measure of global cognitive function • Scientifically validated • Large database of available normative data • Alternate forms for repeated testing • Automated response capture and scoring • Good reliability (ICC: 0.78–0.93) and sensitivity to impairment • Easy to administer and can be scored by non-psychologists • Available in multiple languages	• Computerized version requires patient familiarity with computers
RBANS^82–86^	5 domains; • Immediate memory • Visuospatial/constructional ability • Language • Attention • Delayed memory	Licensing fee; available from Brainworx (✘)	12 RBANS tests^d^: **Outcome measure:** individual task Z-scores and composite score	25:10	Professional staff member	• Assesses 5 domains to yield a measure of global cognitive functioning • Scientifically validated • Good reliability (ICC: 0.84) and sensitivity to cognitive impairment • Easy to administer • Available in multiple languages • Identifies pattern of cognitive impairment	• Requires some training • Profile of patient’s cognitive strengths and weaknesses differs from the MCCB domains
CogState computerized battery^87–91^	7 domains; • Processing speed • Attention/vigilance • Working memory • Verbal learning • Visual learning • Reasoning/problem solving • Social cognition	Licensing fee; available from CogState Ltd (✓)	Customized selection of computerized tasks^e^ **Outcome** **measures:** individual task Z-scores and composite score	20–40:5 (administration times vary depending on number of tests included)	Professional staff member	• Computerized battery with standardized delivery • Available in brief format if all domains not tested • Variable test–retest reliability (ICC: 0.58–0.84) • Sensitivity to cognitive impairment • Established correlations with deficits in MCCB cognitive domains • Assesses 7 cognitive domains to yield global measure of cognitive function • No technical knowledge or training required • Language-independent with translational utility	• Requires patient familiarity with computers
BCA^92^	9 domains; • Processing speed • Attention/vigilance • Verbal learning • Reasoning/problem solving	TMT and VFT are free; Licensing fee for HVLT-R (✘)	3 tests: VFT (letters and categories), TMT parts A and B, and HVLT-R **Outcome** **measures:** individual task Z-scores and composite score	12:3	Professional staff member	• Relatively quick and easy to administer, score, and interpret • Good test–retest reliability (ICC: 0.82) • Established inter-item consistency • Sensitive to treatment effects • Minimal training required • Databases of normative data facilitate comparison of individual scores with reference groups	• Lacks breadth of cognitive assessment provided by full assessment batteries • Too few domains to establish profile of deficits
Penn CNB^93–96^	9 domains • Abstraction and mental flexibility • Attention • Working memory • Episodic memory • Language reasoning • Spatial processing • Sensorimotor • Motor speed • Emotion identification (social cognition)	Free and publicly available (✓)	A collection of computerized tasks that measure neurocognitive functions **Outcome** **measures:** individual task speed/accuracy Z-scores and composite score	60:5 (administration times vary depending on number of tests included)	Professional staff members	• Computerized battery with standardized delivery • Tests based on neurobehavioral functions associated with established brain-systems • Moderate to high reliability depending on the test (Cronbachs Alpha coefficients 0.55–0.98) • Good construct validity • Sensitive to gender and age effects • Minimal training required	• Requires patient familiarity with computers
NIH Toolbox^97,98^	6 (cognitive) domains • Executive function • Attention • Working memory • Episodic memory • Language • Processing speed	Free and publicly available (✓)	Compilation of 47 computerized tasks to assess functioning of individuals across their life span **Outcome** **measures:** individual task Z-scores and composite score	120:5 (administration times vary depending on number of tests included)	Professional staff members	• Good reliability (ICC: 0.78–0.99) • Sensitive to developmental changes • Sensitive to racial, gender and age effects • Applicable across a diverse clinical population and general population	• Originally developed for research use • Requires optimization for clinical utilization

*Note:* BACS, Brief Assessment of Cognition in Schizophrenia; BCA, Brief Cognitive Assessment; CANTAB, Cambridge Neuropsychological Test Automated Battery; CNB, Computerized Neurocognitive Battery; HVLT-R, Hopkins Verbal Learning Test-Revised; MSCEIT, Mayer–Salovey–Caruso Emotional Intelligence Test; NIH, National Institutes of Health; RBANS, Repeatable Battery for the Assessment of Neuropsychological Status; TMT, Trail Making Test; VFT, Verbal Fluency Test.

^a^For computerized assessments, score calculation time is estimated based on author experience at approximately 3–10 min depending on the test.

^b^Individual tests (completion times) consist of Reaction Time, Paired Associates Learning, One Touch Stockings of Cambridge, Multitasking Test, Rapid Visual Information Processing, Emotion Recognition Task, Spatial Working Memory SWM, and Verbal Recognition Memory.

^c^Individual tests consist of Verbal Memory, Digit Sequencing, Token Motor Task, Semantic Fluency & Letter Fluency Tasks, Symbol Coding, and Tower of London.

^d^Individual tests include assessments for list learning, story immediate memory, figure copy, line orientation, picture naming, semantic fluency, digit span, coding, list recall, list recognition, story delayed recall, and figure recall.

^e^Individual test consist of Behavioral Pattern Separation Object Test, Continuous Paired Associate Learning Test, Detection Test, Face Name Associative Memory Exam, Finger Tapping Test, Groton Maze Learning Test, Identification Test, International Daily Symbol Substitution Test—Medicines, International Digit Symbol Substitution Test—Symbols, International Shopping List Test, One Back Test, One Card Learning Test, Psychomotor Vigilance Test, Social-Emotional Cognition Test, Sustained Attention Test, Sustained Attention to Response Test, and Two Back Test.

### Cognitive Assessments of Short Duration (MCCB Subtests; <20 Min Administration Time)

The individual subtests within cognitive batteries such as the MCCB that assess a narrower range of cognitive domains than the overall composite scores may be useful for strategic monitoring of treatments in the clinic. For example, a priori knowledge of specific treatment targets and outcomes may justify focus on particular cognitive domains when assessing treatment effects. These subsets of cognitive assessment batteries have the advantage of being quick and often easier to administer, score, and interpret.

In addition to simplification and shortening of cognitive assessments, the transfer of assessments to online and digital formats also may facilitate access, efficiency, and ease of use for patients.^99^ As neuropsychological assessments are traditionally comprised of interview- and performance-based cognitive assessments, the transition to remote and digital delivery methods is challenging. Spurred by the recent COVID-19 pandemic, remote delivery of cognitive assessments has gained momentum in both research and clinical settings, with varied findings. Remote administration of the Animal Fluency Task (2-min administration time) that provides a measure of verbal fluency (impaired in patients with schizophrenia)^100^ has been shown to be unaffected by mode of administration (in person vs remote) in patients with schizophrenia spectrum disorders and bipolar disorder.^101^ In contrast, remote administration via telephone of the Hopkins Verbal Learning Test-Revised (HVLT-R; a 4-min word list task) was negatively impacted compared with in-person administration, suggesting that in-person normative data may not apply to remote assessments.^101^

Incorporation of touch-screen formats has enabled the characterization of multiple between- and within-test metrics of Trail Making Test (TMT) performance, thus providing greater appreciation of cognitive impairments than the traditional method of scoring. Both the TMT and the BACS-Symbol Coding task that measure speed of processing have been correlated with social function in patients with schizophrenia.^102,103^ Another brief computerized measure, the Continuous Performance Test—Identical Pairs task (CPT-IP), provides a sensitive and reliable measure of attention in healthy individuals and patients.^104–109^ While small practice effects over repeated assessments would need consideration,^106,108^ CPT-IP total score exhibits excellent test–retest reliability and may be suited to assessment of sustained attention in clinical settings.^106^

Deficits in working memory processing can impact on higher cognitive functioning in schizophrenia^110^ and are predictive of functional outcome,^111^ highlighting its importance as a focus for cognitive assessment. Short-term spatial memory is reported to correlate directly with genetic predisposition to schizophrenia, suggesting this is a heritable trait (endophenotype) for schizophrenia.^112^ The Spatial Span from the Wechsler Memory Scale—revision 3 (WMS-III: Spatial Span) is recommended for the assessment of spatial working memory, which refers to the faculty of temporarily encoding, storing, and retrieving visuospatial information for adaptive use that is impaired in patients with schizophrenia.^113,114^ This assessment has been shown to detect age-related decline in spatial working memory^115^ and specific cognitive deficits across a range of psychotic proband groups and in their first-degree relatives.^116^ Paralleling the WMS-III Spatial Span for verbal working memory is the letter–number span test, requiring only 6 min to administer.^117,118^ Computerized versions of both the letter–number and spatial span tests have been developed as part of the Cambridge Neuropsychological Test Automated Battery (CANTAB) assessment and facilitate delivery and scoring of these assessments.^119^

### Cognitive Assessments of Intermediate Duration (20–40 Min Administration Time)

#### The BACS: 30–35 Min Administration Time

The BACS has little additional time needed for scoring and minimal training requirements.^79^ This test is portable and easy to use, yielding high test–retest reliability and completion rates in patients.^79–81^ The BACS includes six tests that assess four of the most consistently-affected cognitive domains (Table 3).^79^ A digital version of the BACS for tablet-based delivery (BAC App) has been developed, allowing standardized administration, reduced rater-related error variance, and more efficient automated scoring.^120^ The BACS is able to assess aspects of cognition that correlate with important everyday functioning measures in clinical trials of cognitive enhancement^80^ and has been validated in a number of languages.^81,121–126^ This assessment was as sensitive to global cognitive change following treatment as the more time-intensive Clinical Antipsychotic Trials of Intervention Effectiveness (CATIE) neuropsychological battery in patients with schizophrenia,^127^ supporting the potential usefulness of this abbreviated cognitive battery in clinical contexts.

#### Repeatable Battery for the Assessment of Neuropsychological Status (RBANS): 25–35 Min Administration Time

The RBANS was designed as an abbreviated cognitive screening tool that could be utilized by professionals with varying levels of training and experience.^83^ Similar to the previous cognitive assessment batteries, the RBANS produces reliable and valid measures of global cognitive functioning that correlate well with overall scores from comprehensive batteries.^83,128,129^ The RBANS provides valuable information about the pattern of cognitive alterations in patients, correlates significantly with standard measures of intelligence and memory, is largely independent of symptom severity, and has been validated as a useful screening assessment of cognitive impairments in patients with schizophrenia and adolescents with psychotic symptoms.^82,84^

#### CogState Computerized Battery: 20–40 Min Administration Time

The CogState computerized battery represents a standard computerized assessment that was created as a non-language-based alternative to the MCCB with similar test–retest reliability^87^; however, MCCB domains correlate better with social skills performance, presenting a potential advantage over the CogState in the measurement of cognitive functioning.^88^

## Social Cognitive Assessments

Reduced social motivation, misinterpretations of the social intent of others, and impaired ability to develop social relationships, can contribute significantly to poor daily functioning in schizophrenia. In contrast to non-social cognition, current assessments for this domain are not as well-established or validated,^6^ and have been hindered by a lack of consensus regarding optimal measurement strategies and methodologies for establishing validity.^6^ Initiatives to develop improved tests of emotion processing include an emotion processing battery with a large normative sample, the Mayer-Salovey-Caruso Emotional Intelligence Test (MSCEIT).^130^ The MSCEIT has demonstrated reliability in measuring social cognitive impairments in patients with schizophrenia that are meaningfully related to measures of neurocognitive function and psychopathology.^130^ The Penn Computerized Neurocognitive Battery, which was created using tests validated with functional neuroimaging to assess performance in neurobehavioral domains, also offers a reliable means of measuring social cognition.^93,94^ Specifically, the Penn Emotion Identification Test measures a person’s ability to decode and correctly identify facial expressions of emotion.^93^

A more recent abbreviated assessment battery, with an estimated administration time of 15 min, was developed as part of the Brief Battery of the Social Cognition Psychometric Evaluation study (BB-SCOPE) to facilitate measurement of social cognition in individuals with schizophrenia spectrum disorders.^131^ The BB-SCOPE battery comprehensively assesses three domains of social cognition (ie, attributional bias, emotion processing, and theory of mind), has sufficient sensitivity to detect social impairments in patients with schizophrenia spectrum disorders, with the advantage of a simple scoring method.^131^ An alternative approach is provided by the Observable Social Cognition: A Rating Scale (OSCARS), which incorporates informant ratings in the assessment of social cognition and also requires only 15–20 min to administer.^132^ In patients with schizophrenia, the OSCARS demonstrated psychometric reliability, modest evidence of convergent validity, and significant correlations with measures of functional outcome and neurocognition.^132,133^ The OSCARS is a potentially useful, brief, and easily implemented clinical screening tool to detect impairment in social cognition, but informant availability is an important consideration.^133^ Further development and validation of brief formats for assessing changes in diverse aspects of social cognition, while also being easily accessed and administrated, are needed to increase their utilization in clinical settings, and ultimately assist in aligning patients with optimal treatments.

## Benefits and Challenges of Current Assessments

The capacity to assess the relevant cognitive domain(s) across different geographical, economic, and cultural contexts is an important consideration for global application of assessments in real-world clinical settings.^134^ Larger assessment batteries such as the MCCB and interview-based assessments such as the SCoRS have been translated into many different languages and successfully validated across different countries,^8,65,66,135^ allowing wider application of these more comprehensive assessments. Due to the broad scope of assessments such as the MCCB and CANTAB, these neuropsychological batteries may also be adaptable for screening and categorization of patients into cognitive subgroups, which in the future may allow for potential refinement of schizophrenia endophenotypes, mapping of specific biological mechanisms, and tailored clinical treatments in many clinical settings.^136,137^ This subtyping of patients based on their cognitive impairments has been performed more recently using the BACS, suggesting that more abbreviated batteries can be equally as sensitive to impairments in specific MATRICS cognitive domains.^138^ Despite the benefits of these validated full cognitive test batteries, they are more suited to large scale clinical trials that have the trained clinical research staff, external funding, and time to successfully implement, than they are for typical clinical contexts. In a consensus meeting discussing issues relating to clinical cognitive assessments in schizophrenia, a divergence of opinion was evident between clinicians and research psychologists in relation to the practicality versus the validity and usefulness of shorter formal assessments for this purpose.^128^ Clinicians with limited time and access to resources generally advocated for the use of shorter assessments such as the BACS and RBANS and interview-based techniques that are more realistically implemented. However, research psychologists with concerns about the psychometric characteristics and validity of these assessments questioned the value and quality of the data provided by brief assessments that do not adequately capture the breadth and complexity of cognitive deficits.^128^ The assessments that ranked the highest among clinicians and researchers in terms of their value for application in clinical settings were brief (15–30 min) cognitive performance assessments, performance-based measures of functional capacity, briefer (5–10 min) cognitive performance assessments, and interview-based measures of cognition and functioning. These valuable discussions emphasize the need to develop shorter validated cognitive assessments with strong test–retest reliability, limited practice effects, and demonstrated relationships with everyday functioning. This development may require an additional consensus process which includes clinicians from community clinics, and considers studies that validate brief or self-administered instruments and trials with real-world implementation.

The translatability and capacity to adapt assessments for use across diverse cultures and global populations is an essential consideration that can extend the reach and enhance consistency and comparability of cognitive assessments.^67^ The Cross Cultural Adaptability of Intermediate Measures Study is a MATRICS initiative that surveyed international clinical trial experts in schizophrenia to evaluate the cultural adaptability of functional capacity and interview-based assessments by country.^70^ The CAI assessment was rated as the most easily adapted and appropriate for cross-cultural administration of intermediate measures of cognitive functioning.^67,70^ Despite weaker correlations of CAI to the MCCB,^59^ this interview-based assessment may be beneficial as a supplement to brief performance measures in different cultures and languages.

With shorter completion times, the validated RBANS and BACS may currently represent the best-suited assessment tools for typical clinical applications,^79,81,82,84,121–126^ yet even with 25–30-min administration times, significant challenges exist for mental health services that may not have staff with the specialized training or time required for these assessments or access to reimbursements.^128^ There is an unmet need to develop and validate cognitive assessments that are shorter in duration while still providing adequately sensitive, psychometrically sound, easy to administer/score, cost-effective and culturally appropriate measures of cognitive performance.

Reducing the time required to complete assessments is desirable from the point of view of busy clinical institutions to maximize cost efficiency, as staff turnover can reduce the availability of trained staff. Also, from the patient perspective, assessments of longer duration, during visits to the clinic, which may already take several hours, can impact negatively on patient engagement and completion rates. Assessments that are shorter in duration than the BACS include the 15-min Brief Cognitive Assessment (BCA),^92^ and the 10-min Brief Cognitive Assessment Tool for Schizophrenia (B-CATS) tests.^73^ One such assessment in development is the cognition self-assessment rating scale (C-SARS) which is a very brief self-report based cognitive test that has been adapted for remote online use.^139^ Although these brief cognitive assessment batteries may provide adequate measures of global cognitive function in schizophrenia spectrum disorders, one major disadvantage is that they are not as sensitive as larger test batteries to improvement/decline in specific cognitive domains that may occur during the course of illness or treatment.^73,79,92^ Furthermore, despite the lower costs associated with brief assessments such as the BCA, BACS, and B-CATS relative to larger batteries of tests, there is still a requirement for trained professional staff time in their implementation, scoring, and interpretation, along with the complexity relating to administration. These factors, combined with possible limitations for reimbursement of these assessments, decreases the likelihood of their use in busy clinical practices. However, as effective treatments for cognitive deficits in schizophrenia become available, clinicians may need to adapt to the need for occasional cognitive assessments to monitor improvements. In the same way that blood assays, which are associated with time and expense, are conducted to monitor metabolic changes during treatment with antipsychotics, brief cognitive measurements may require similar prioritization in the future.

## Future Opportunities

The integration of research findings correlating specific cognitive impairments with biological measures is crucial to facilitate targeted treatments in the clinic; for example, neuroimaging, neurochemical, physiological and/or genomic biomarkers, and other behavioral changes. Linking biomarkers of neuropathology from neuroimaging studies with specific cognitive impairments identified from clinical assessments would likely enhance understanding of the neurobiological bases of CIAS, facilitate the identification of cognitive subtypes within the spectrum of impairments that exist in the CIAS syndrome, and allow for more targeted treatment of CIAS. With this aim, the Cognitive Neuroscience Test Reliability and Clinical Applications for Schizophrenia (CNTRaCS) Consortium was established.^140^ Reliability of CNTRaCS tasks in the measurement of discrete cognitive abilities, and modest correlations with functional outcomes have been demonstrated in patients with schizophrenia.^129^ The current focus of CNTRaCS is the increased utilization of computational modeling to identify measures that correlate with specific cognitive and visual processes that could enhance understanding of discrete and shared pathophysiological mechanisms across cognitive disorders.^140^ Although this initiative will not have an immediate impact on the optimization of cognitive assessments for clinical practice, it may in the future suggest ways to use computational and technology-based assessments in the clinic.

Digital technologies in cognitive assessment can be used to convert data-poor clinical endpoints associated with neuropsychiatric disease assessment into a richer, scalable, and objective set of measurements.^141,142^ Computerized assessments have already been developed and applied to provide measures of cognitive function. Adaptation for web-based cognitive assessment could improve patient access, thus broadening the reach among the patient population, and increase the flexibility of application in clinical settings.^143^ With the remarkable uptake of digital devices, measurement of cognition can be adapted for settings outside the clinic, and may prove useful in monitoring and treating CIAS.^142,144^ However, differences in context may influence task performance and caution is recommended when interpreting web-based versus in-person assessments.^143^

Prompted by an increased need for more accessible platforms during the COVID-19 pandemic (2020–2022), greater focus has been placed on the validation of online delivery methods for cognitive assessments. Recent studies have demonstrated that certain neuropsychological assessments may be amenable to remote administration using technology-based approaches that allow a broader capture of cognitive responses in patients with schizophrenia spectrum disorders.^99,120,145–147^

Currently, a number of cost-related and logistical challenges prevent cognitive profiling on a larger scale to be routinely implemented in clinical settings. Therefore, there is a growing need for reliable and valid evaluative tools to assess cognition that can be administered and interpreted easily and are adaptable for remote settings, minimizing administration, and reducing the need for specially-trained clinical personnel while also increasing patient access. Progress in these directions is evident in recent research. A study examining the validity of remote administration in older adults of four MCCB tests measuring processing speed (TMT: Part A, Animal Fluency), working memory (Letter–Number Span), and verbal learning and memory (HVLT-R) revealed that although performance on some tests was significantly affected by administration format, remote administration of other MCCB subtests may provide a valid alternative to in-person testing.^101^ Similarly, a tablet version of the BACS (BAC App) administered by a trained rater at a research site revealed a high level of feasibility and reliability, demonstrating equivalence between tablet and paper-and-pencil versions.^120^ Additionally, the BAC App was recently adapted for remote self-administration in the absence of medical staff supervision, and assessments were limited to four tests. After in-person training on the iPad platform, remote assessment of older adults yielded comparable results to in-person assessment for three of four tests.^148^ If this level of feasibility and comparability can be demonstrated in a schizophrenia sample, this tablet battery would appear appropriate for monitoring cognitive change in clinical practice.

In the absence of trained staff supervision and control over the testing environment, performance on remote cognitive assessments can be influenced by numerous environmental and symptom-related factors, so there is a need for further performance validity testing to establish the conditions under which accurate interpretation by clinicians can occur.^95,142^ A recent review revealed that despite their potential for remote assessment, the computerized cognitive batteries, CANTAB and CogState, have not been utilized extensively in remote settings.^142^ However, other computerized comprehensive batteries with the potential for remote administration were evaluated for their psychometric properties.^142^ The Online Neurocognitive Assessments (40 min administration time) measure five cognitive factors, four of which had moderate correlations with corresponding MCCB domains (but not social cognition) when administered in the laboratory.^145^ My Cognition Quotient (30 min administration time) assesses five cognitive factors, three of which correspond adequately with CANTAB cognitive domains when administered in the laboratory.^146^ The Screen for Cognitive Assessment in Psychiatry (15 min administration time) has been administered remotely by videoconferencing in a small study with patients with schizophrenia and was found to have acceptable internal consistency.^147^ Of the five measures evaluated, performance on two was significantly different between videoconference and in-person administration. Further research involving remote administration of these computerized batteries is needed to directly address whether remote administration alters performance levels. A limitation of most of these computerized measures at present is the lack of test–retest reliability and normative data based on remote assessment,^142^ making these important aspects for future development. Another significant barrier to widespread application of available cognitive assessment batteries in remote formats is that they are often proprietary, and therefore involve significant costs and limited flexibility for customized use. The Inquisit platform provides a mechanism for developing remote psychological testing across multiple geographical regions and offers an alternative remote method for cognitive data collection without requiring in-person physical attendance.^149^ This platform has demonstrated reliability equivalent to other laboratory-based platforms (MATLAB, Psychtoolbox extension) for some measures, and comparable results to the CANTAB supervised computerized battery in healthy volunteers,^150,151^ providing significant advantages relating to the scalability and broader reach when compared with in-person assessments. Inquisit does require purchasing a license and has not yet been used to develop a wide range of neuropsychological measures. Further work is also required to confirm whether the normative data sets used for interpretation are appropriate for remote testing.

A hybrid neuropsychology model combining both traditional and technology-based modalities has recently been proposed that facilitates the integration of data science into the clinic and promotes collaboration with experts in other fields.^99^ This amalgamation of assessment approaches may represent a key initial step in the transition to greater utilization by clinicians of technology-based assessments for cognitive profiling of patients that could be implemented with greater ease in both in-patient and out-patient settings.^99^ However, further studies to evaluate the validity of technology-based and remote assessments for cognition are required, and essential clinician training is needed, to facilitate this transition. Typically, board-certified neuropsychologists spend 2–5 years receiving neuropsychology-focused training that includes theoretical background, training in neurological and neuropsychiatric syndromes, and also training in the administration, scoring, and interpretation of neuropsychological assessments.^99^ Broadening the application of cognitive assessments in patients experiencing schizophrenia may require consideration of alternative training models for clinicians that focus specifically on a narrower range of measures, with emphasis on emerging technology-based assessments. The type and level of clinician training and the oversight of test result interpretation will differ from one cognitive measure to the next, so cognitive test developers will need to address training requirements as part of their test distribution. The provision of Continuing Medical Education credits in association with these more focused training initiatives may facilitate their broader dissemination and implementation in the clinic.

Other considerations for the use of remote assessments with existing cognitive measures include determining if the normative data from in-person administrations of these assessments are accurate for remote assessments and establishing optimal test settings (eg, a quiet room free from interruptions). Considering that testing conditions for these assessments are normally tightly controlled during in-person administration, the impact of varying testing conditions on results needs more examination. Thus, further investigation of remote assessments for cognitive impairments is essential to ensure their validity and determine their comparability to in-person assessments. Logistical issues that are essential to maximize accessibility and quality of remote cognitive assessments include the standardization of methods, mitigation of potential issues of internet connectivity, the choice of platform (smartphone, internet, videoconferences) that have diverse functionality and potentially impact patient performance and acceptability.^142^ In addition, ethical considerations are also important to ensure security and privacy of collected patient data that will impact on patient acceptability of remote assessments.^142^

Consideration of patient and caregiver perspectives in the development of future approaches is important to optimally engage patients and create more patient-centered clinical assessments. Collecting and evaluating caregiver and patient opinions, attitudes, and perspectives help to inform assessment design, delivery and interpretation, and identify areas for improvement.^152^ For example, brief assessments such as the recently developed C-SARS incorporate patient self-reports of cognition that reflect measures of daily functioning, thus emphasizing the patient perspective.^139^ Similarly, the CAI and SCoRS assessments capture informant evaluations of cognitive functioning that contribute valuable insight into cognitive impairments.^54,61^

## Conclusions

The past two decades have seen an increase in the focus of clinical research to improve understanding of cognitive deficits in schizophrenia, and development of new and effective non-pharmacologic cognitive therapies for CIAS. Consequently, there exists a strong need to establish reliable and consistent approaches to the assessment of cognitive impairments in clinical settings. Current validated assessments present several challenges for successful and consistent implementation in typical clinical settings, including costs associated with training, staff time for the administering and scoring of assessments, and additional infrastructure. There is a need to establish improved assessment formats that have comparable sensitivity to traditional batteries in detecting cognitive improvement/decline in patients, while also minimizing the staff time and training necessitated in the delivery of these assessments. A key initial step in advancing cognitive assessment in clinical practice would be to develop a toolkit similar to the OnTrackNY recently evaluated in patients with early psychosis, that equips clinicians with the necessary guidance to identify optimal approaches to assessing and monitoring cognitive impairments for individual patients within the confines of clinical settings.

**Fig. 1.**
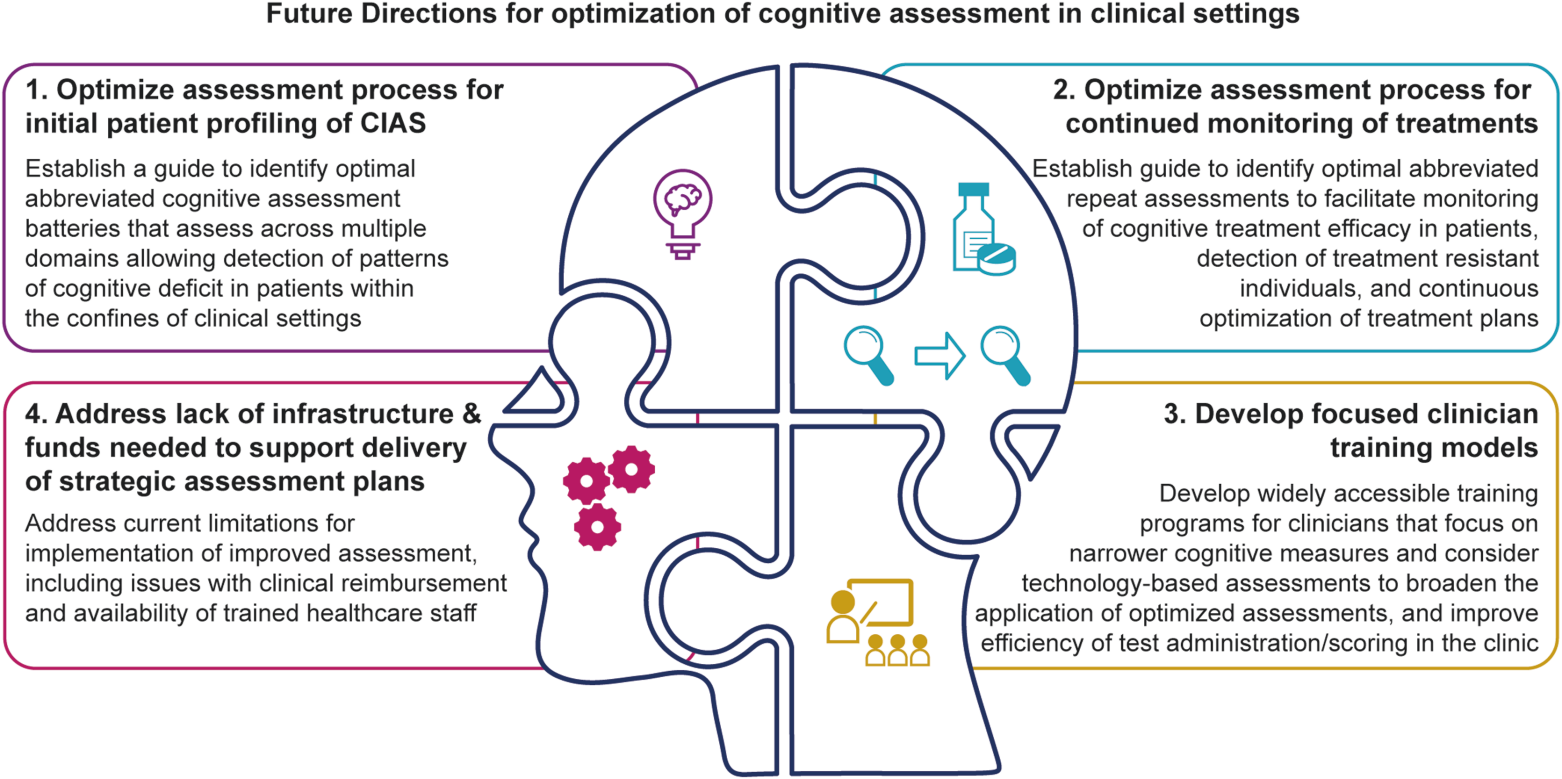
A diagram summarizing proposed next steps for the optimization and delivery of improved cognitive assessment in clinical settings. *Note:* CIAS, cognitive impairment in schizophrenia.

Initial profiling of cognitive deficits in patients may require longer assessment batteries (>30 min) that span multiple cognitive domains, with subsequent monitoring of treatment effects using shorter and more targeted assessments that are amendable to repeated testing (Fig. 1). Although assessments such as the BACS and RBANS provide options that can be completed within a 30-min period, this may not be brief enough for practical application in some typical “real-world” clinic settings. It would be useful to focus on the validation of abbreviated assessments (10–15 min) that reliably measure global cognitive change for follow-up assessment of treatment effects in patients. This strategy is particularly relevant when a priori knowledge of treatment mode-of-action is known. Considering the diverse range of current assessments for cognition in schizophrenia, providing a roadmap for current and novel cognitive assessments would enhance therapeutic decision making for clinicians when addressing CIAS. Additionally, as the potential for incorporation of technology-based and remote assessments in everyday settings increases with improving digital literacy, the need for validation of remote or hybrid formats of cognitive assessments is important. Integration of computerized formats, and the implementation of broader assessments for CIAS in clinical settings, will necessitate the establishment of readily accessible and focused training programs to hone clinician skills and facilitate delivery of assessments in the clinic. Ultimately, the main challenges that exist for clinicians in implementation of effective assessments of CIAS include not only the time required by current validated assessment formats, but also issues relating to clinical reimbursement and availability of trained healthcare personnel. As such, clear guidance on an optimized and cost-effective cognitive assessment process for CIAS, and a strategic focus on the provision of the required training and infrastructure for effective implementation, are essential.
